# The Balance of Th1/Th2 and LAP+Tregs/Th17 Cells Is Crucial for Graft Survival in Allogeneic Corneal Transplantation

**DOI:** 10.1155/2018/5404989

**Published:** 2018-02-08

**Authors:** Shang Li, Jing Yu, Chungang Guo, Ying Jie, Zhiqiang Pan

**Affiliations:** ^1^Beijing Tongren Eye Center, Beijing Tongren Hospital, Capital Medical University, Beijing, China; ^2^Department of Ophthalmology, Beijing YouAn Hospital, Capital Medical University, Beijing, China

## Abstract

**Purpose:**

CD4+LAP+ T cells are newly discovered regulatory T cells (Tregs). The aim of this study is to investigate the balance of Th1/Th2 and LAP+Tregs/Th17 in mice after allogeneic corneal transplantation.

**Methods:**

A total of 65 mice received orthotopic penetrating transplantation. According to the survival scores of the grafts, the mice were divided into the rejection group and the survival group 3 weeks after transplantation. Th1, Th2, Th17, and regulatory T cells in the ipsilateral drainage lymph nodes and spleens were measured with flow cytometry. The related cytokines in aqueous humor were also analyzed.

**Results:**

The frequencies of Foxp3+Tregs, GARP+Tregs, and LAP+Tregs in the survival group were significantly higher than those in the rejection group. And the expression trend of CD4+LAP+ T cells and CD4+GARP+ T cells was consistent. The level of IFN-*γ*, TNF, IL-6, and IL-17A markedly increased in aqueous humor during corneal allograft rejection. The ratio of Th1/Th2 and Th17/LAP+Tregs significantly increased in the rejection group at the 3rd week after corneal transplantation.

**Conclusion:**

LAP+Tregs might be regarded as substitute for Foxp3+Tregs. The balance of Th1/Th2 and LAP+Tregs/Th17 is crucial for corneal allograft survival.

## 1. Introduction

Corneal transplantation is one of the most prevalent and successful forms of solid tissue transplantation in the world [1]. In a cohort study of 10,952 cases, the graft survival rate 1 year after penetrating keratoplasty is as high as 86% [2]. However, in some circumstances such as inflammation, neovascularization, or infections in corneal graft beds, the immune privilege of corneal allografts can be abolished [3]. Despite the local and systemic corticosteroid and immunosuppressive therapy, immune rejection is still the leading cause of corneal allograft failure after penetrating keratoplasty [4].

There are several ways to prolong the survival of the corneal allografts. One of the most important methods is the induction of regulatory T cells to inhibit corneal immune rejection [5]. Foxp3 expressing CD4+CD25+ regulatory T cells play a central role in inducing and maintaining immune tolerance in allogeneic corneal transplantation [6]. But the expression of Foxp3 can also be found in activated effector T cell (Teffs), which limits it as a specific marker for Tregs [7].

In recent years, a study found that activated Tregs could express latency-associated peptide (LAP) on the surface through the anchoring molecule glycoprotein A repetitions predominant (GARP) [8]. Some researchers found although CD4+LAP+ T cells lack Foxp3 expression but they can secrete IL-10 and TGF-*β* upon activation and exhibit immune-suppressive activity in vitro [9]. Oida and Weiner also reported that TGF-*β* can induce LAP expression not only on T cells that converted to Foxp3+ T cells but also on T cells in which Foxp3 was not expressed [10]. Therefore, LAP+Tregs instead of Foxp3+Tregs may play an important role in immune tolerance. In this study, the model of orthotopic penetrating transplantation in mice was used to investigate the imbalance of Th1/Th2 and LAP+Tregs/Th17 in survival and rejection mice, aiming to provide a basis for further study about the role of functional LAP+Tregs in corneal transplantation.

## 2. Materials and Methods

### 2.1. Animals

The animals were provided by the Experimental Center of Capital Medical University, Beijing, China. C57BL/6 (H-2b) mice were used as donors, and BALB/c (H-2d) were used as recipients. All the animals were male, aged 6~8 weeks old, and were housed under specific pathogen-free facility. The use of the animals was ethically approved by the Animal Care and Research Committee of Capital Medical University.

### 2.2. Corneal Transplantation

Orthotopic penetrating transplantation was performed as described previously [11]. Briefly, the recipients were anesthetized by intraperitoneal injection with 4‰ pentobarbital sodium. The allografts of 2 mm diameter from the donor corneas were placed in the recipient bed with the same size and then secured with eight interrupted 11-0 nylon sutures. The sutures were removed 7 days after surgery. Tobramycin and dexamethasone eye ointments were administered only once in the conjunctival sac after surgery, and the eyelids were stitched with 6-0 silk sutures. The eyelid sutures were removed 3 days after surgery.

### 2.3. Assessment of the Grafts

A total of 65 recipient BALB/c mice received surgery, of which 40 mice underwent allogeneic corneal grafts from donor C57BL/6 mice and the other 25 mice received the syngeneic corneal grafts from donor BALB/c mice as control. All recipient mice were examined under a slit lamp biomicroscope three times a week after surgery for 4 weeks. Digital photographs of the cornea were taken using a Nikon D90 camera attached to the slit lamp biomicroscope. The survival of the grafts was defined according to a previously reported scoring system: 0, clear graft; 1+, minimal superficial opacity; 2+, minimal deep stromal opacity with pupil margin and iris vessels visible; 3+, moderate deep stromal opacity with only the pupil margin visible; 4+, intense deep stromal opacity with the anterior chamber visible; and 5+, maximum stromal opacity with total obscuration of the anterior chamber. Grafts with opacity scores of 2+ or greater after 3 weeks were considered to have been rejected [12].

### 2.4. Histopathology

Five recipient mice in each group were sacrificed, and the eyeballs were taken off on day 21 after surgery. Then the corneas were fixed in 10% formaldehyde solution and embedded in paraffin wax. The embedded tissue was sliced to 5 *μ*m thickness and stained with hematoxylin eosin.

### 2.5. Flow Cytometric Analysis

Ipsilateral draining submandibular lymph nodes, cervical lymph nodes, and spleens were harvested from the mice before surgery and at the 1st week, 2nd week, 3rd week, and 4th week after surgery. The single cell suspension from the spleens and lymph nodes was centrifuged, and the final cell concentration was adjusted to 2 × 10^7^/mL. Each sample was then divided into two parts. The 100 *μ*L cell suspension was taken to the bottom of the flow tube and incubated with the anti-mouse CD4-FITC, anti-mouse GARP APC, and anti-mouse LAP PerCP-Cy5.5 (eBioscience, CA, USA) at 4°C for at least 30 minutes in the dark and then washed, fixed, permeabilized, and stained with anti-mouse Foxp3-PE for another 30 min at 4°C in the dark. The other cell samples were resuspended in RPMI 1640 media supplemented with 10% FBS, antibiotics, and L-glutamine and stimulated with 50 ng/mL PMA and 500 ng/mL ionomycin for 6 h, the last 2 h with the addition of GolgiPlug (BD Biosciences, CA, USA) at 37°C. Following that, they were incubated with anti-mouse CD4-FITC for at least 30 min at 4°C, fixed with fixation/permeabilization solution, and stained with anti-mouse IL-10 PE, anti-mouse IFN-*γ* APC, anti-mouse IL-4 PE-Cy7, and anti-mouse IL-17A PerCP-Cy5.5 for another 30 min at 4°C in the dark. Finally, the cells were resuspended with the flow cytometry staining buffer and analyzed using a BD FACS LSRII flow cytometer (BD Biosciences, CA, USA) and Cell Quest software.

### 2.6. CBA

The aqueous humor samples were collected using a microinjector (Hamilton 1710 RN, SYR 100 *μ*L, Swiss). The samples were centrifuged at 4°C for 3 min and then stored at −80°C. Cytokines in the aqueous humor samples were measured with BD CBA Mouse Th1/Th2/Th17 Cytokine Kit as previously described (BD Bioscience, CA, USA) [13]. The kit was used for the simultaneous detection of mouse interleukin-2 (IL-2), interleukin-4 (IL-4), interleukin-6 (IL-6), interferon-*γ* (IFN-*γ*), tumor necrosis factor (TNF), interleukin-17A (IL-17A), and interleukin-10 (IL-10) in a single sample. This array kit provides a mixture of seven capture beads with distinct fluorescent intensities that have been coated with capture antibodies specific for each cytokine. The operations were performed according to the manufacturer's instruction. The beads coated with seven specific capture antibodies were mixed, and then the 5 *μ*L aqueous humor sample was diluted to 50 *μ*L by the desired dilution factor (1 : 10) using the appropriate volume of Assay Diluent. Subsequently, 50 *μ*L mixed captured beads, 50 *μ*L standard dilutions, and 50 *μ*L phycoerythrin (PE) detection reagent were added consecutively to each assay tube and incubated for 2 h at room temperature in the dark. The samples were washed with 1 mL wash buffer (200 g) for 5 min and then centrifuged. The bead pellet was resuspended in 300 *μ*L buffer after discarding the supernatant. In the end, the samples were measured on the BD FACS LSRII Flow Cytometer and analyzed by FCAP Array™ software (BD Bioscience). The theoretical limits of detection were 0.1 pg/mL for IL-2, 0.03 pg/mL for IL-4, 1.4 pg/mL for IL-6, 0.5 pg/mL for IFN-*γ*, 0.9 pg/mL for TNF, 0.8 pg/mL for IL-17A, and 16.8 pg/mL for IL-10.

### 2.7. Statistical Analysis

Statistical analysis was performed using SPSS (21.0, SPSS Inc., IBM Corporation, New York, USA). All data are presented as mean ± standard deviations. The survival curves of corneal grafts in different groups of mice were established by Kaplan-Meier analysis and comparisons between groups by log-rank analysis. The difference in the frequencies of T cells and cytokine levels was analyzed by univariate analysis of variance. Values of *P* < 0.05 were considered statistically significant. All photographs were performed using GraphPad Prism 5.0 software (GraphPad software, USA).

## 3. Result

### 3.1. Survival Time of Corneal Graft and Histopathology

**Figure 1:**
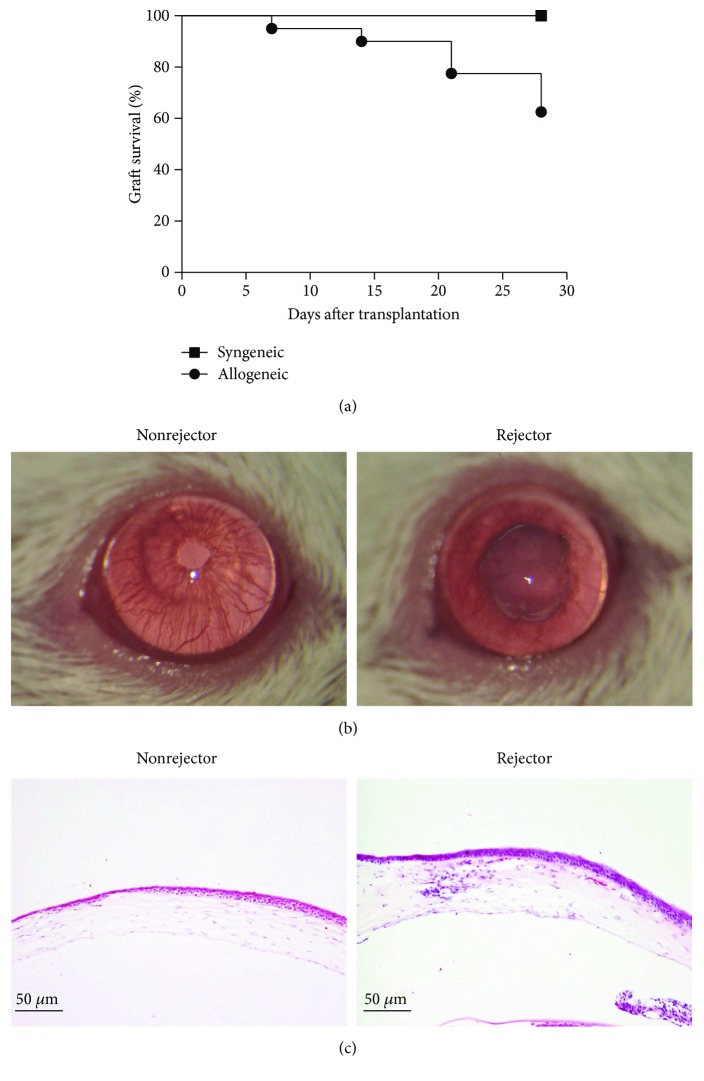
Survival of the grafts after corneal transplantation. (a) Kaplan-Meier survival curve showed that 15 of 40 allogeneic corneal grafts underwent acute rejection 4 weeks after transplantation, while there was no rejection in all syngeneic grafts. (b) The grafts were clear in the survival group while the grafts were severe opacity in the rejection group (magnification ×16). (c) Histopathology results showed that there was no edema and inflammatory cell infiltration in the survival group, while there was severe edema, inflammatory cell infiltration, and neovascularization in the rejection group (magnification ×10, bar = 50 *μ*m).

Approximately 37.5% (15 of 40) of the grafts in the allogeneic group underwent acute rejection 4 weeks after transplantation, while no graft in the syngeneic group was rejected (Figure 1(a)). There was a significant difference of survival time between the two groups (*χ*^2^ = 9.341, *P* = 0.002). The grafts were clear, and the vessels of the iris were also clearly seen in the survival group, while the grafts were severe opacity and edema in the rejection group and the details of the iris could not be seen (Figure 1(b)). The pathological examination further showed that the thickness of the grafts was normal, and there were no inflammatory cells or neovascular blood vessels in the stroma in the survival group. But in the rejection group, the thickness of the grafts was significantly increased and there was some inflammatory cell infiltration with neovascularization in the stroma (Figure 1(c)). The quantitative analysis of inflammatory cells in the tissue is presented in Table 1.

### 3.2. Flow Cytometric Analysis of T Cells in Secondary Lymphoid Organs

**Figure 2:**
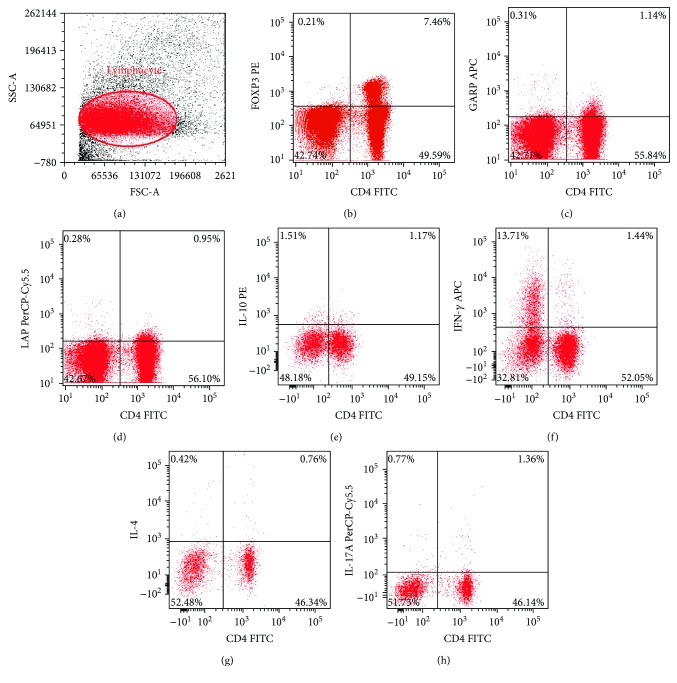
Gating strategy is represented for each cell type assessed using flow cytometry. Representative data of lymphocyte, CD4+Foxp3+ T cells, CD4+GARP+ T cells, CD4+LAP+ T cells, CD4+IL-10+ T cells, CD4+IFN-*γ*+T, CD4+IL-4+ T cells, and CD4+IL-17A+ T cells in the lymph nodes at the 1st week after corneal transplantation (a–h).

**Figure 3:**
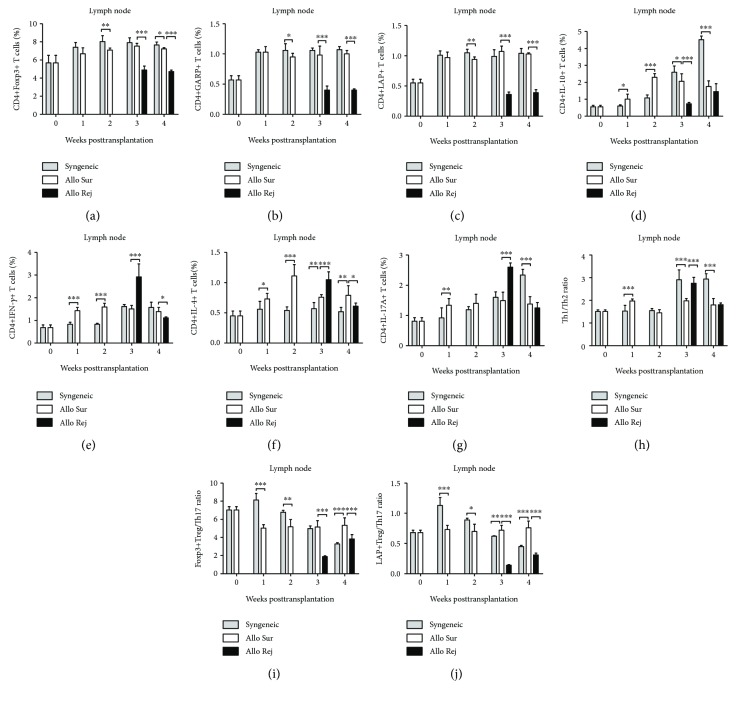
Assays of T cells from draining lymph nodes (LN) after corneal transplantation. (a–g) The numbers of CD4+Foxp3+ T cells, CD4+GARP+ T cells, CD4+LAP+ T cells, CD4+IL-10+ T cells, CD4+IFN-*γ*+T, CD4+IL-4+ T cells, and CD4+IL-17A+ T cells from the survival group (Allo Sur), the rejection group (Allo Rej), and the syngeneic group (Syn) were analyzed by flow cytometry. (h–j) The ratio of Th1/Th2 and Tregs/Th17 was calculated at each time point. *n* = 5 in each group. The data are presented as the mean + SEM; ^∗^*P* < 0.05, ^∗∗^*P* < 0.01, and ^∗∗∗^*P* < 0.001. Th: T helper; Tregs: regulatory T cells.

**Figure 4:**
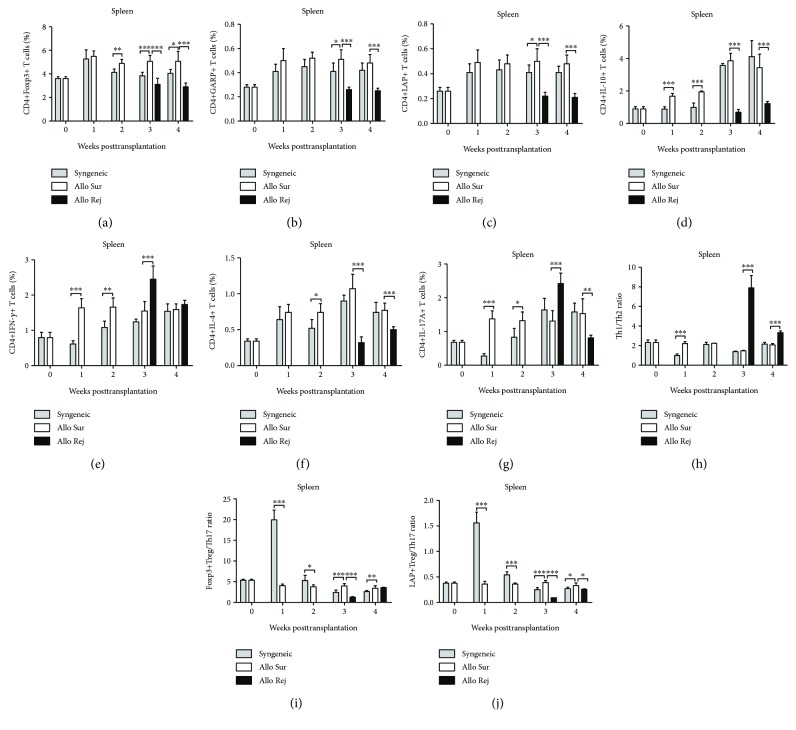
Assays of T cells in the spleen (SP) after corneal transplantation. (a–g) The number of CD4+Foxp3+ T cells, CD4+GARP+ T cells, CD4+LAP+ T cells, CD4+IL-10+ T cells, CD4+IFN-*γ*+T, CD4+IL-4+ T cells, and CD4+IL-17A+ T cells from the survival group (Allo Sur), rejection group (Allo Rej), and syngeneic (Syn) group were analyzed by flow cytometry. (h–j) The ratio of Th1/Th2 and Tregs/Th17 was calculated at each time point. *n* = 5 in each group. The data are presented as the mean + SEM; ^∗^*P* < 0.05, ^∗∗^*P* < 0.01, and ^∗∗∗^*P* < 0.001. Th: T helper; Tregs: regulatory T cells.

We investigated the T lymphocyte subset count using flow cytometry (Figures 2(a)–2(h)). The frequencies of CD4+Foxp3+ T cells, CD4+GARP+ T cells, and CD4+LAP+ T cells in the lymph nodes and spleens were increased at the 1st week after transplantation compared with the baseline in both syngeneic and allogeneic mice, and the concentration remained at a higher level during the whole 4 weeks. The frequencies of CD4+Foxp3+ T cells, CD4+GARP+ T cells, and CD4+LAP+ T cells in the rejection group were significantly decreased at the 3rd week and 4th week after transplantation compared with the survival group. Furthermore, the frequencies of these three regulatory T cells in the syngeneic group were higher than those in the allogeneic group at the 2nd week, but there was no statistical difference at the 1st, 3rd, and 4th week between these two groups in the lymph nodes (*P* > 0.05) (Figures 3(a)–3(c) and 4(a)–4(c)). The frequency of CD4+IL-10+ T cells was increased, respectively, in the allogeneic group and syngeneic group from the 1st week to 2nd week after surgery. In the first 2 weeks, the frequency of CD4+IL-10+ T cells in the syngeneic group was lower than that in the allogeneic group, but higher than that in the allogeneic group from the 3rd week after surgery in the lymph nodes. There was a statistically significant difference in the frequency of CD4+IL-10+ T cells between the survival and the rejection group at the 3rd week (*P* ≤ 0.001), but there was no difference at the 4th week between these two groups in the lymph nodes (*P* = 0.211). However, there was an obvious decrease in the rejection group in the spleens at the 4th week (Figures 3(d) and 4(d)). In the first 2 weeks, the frequency of CD4+IFN-*γ*+ T cells in the syngeneic group was lower than that in the allogeneic group, but increased to the same level of the allogeneic group at the 3rd week. The frequency of CD4+IFN-*γ*+ T cells at the 3rd week in the rejection group was 2.92 ± 0.57% in the lymph nodes and 2.54 ± 0.37% in the spleens, significantly higher than that in the survival group. Then the frequency decreased to 1.11 ± 0.05% in the lymph nodes and 1.73 ± 0.12% in the spleens at the 4th week (Figures 3(e) and 4(e)). There was no significant increase in the frequency of CD4+IL-4+ T cells in the syngeneic group during the whole observation period. However, the frequency of CD4+IL-4+ T cells in the allogeneic group was higher than that in the syngeneic group in the lymph nodes at each time point. The frequency of CD4+IL-4+ T cells in the rejection group 3 weeks after transplantation abnormally elevated significantly (*P* = 0.001) and then decreased to a lower level than that in the survival group at the 4th week in the lymph nodes (*P* = 0.02) (Figure 3(f)). In the spleens, the frequency of CD4+IL-4+ T cells increased gradually in both the syngeneic and the allogeneic group, but kept an even lower level in the rejection group at the 3rd week (Figure 4(f)). The frequency of CD4+IL-17A+ T cells was increased in both the syngeneic and the allogeneic groups, from the 2nd week in the lymph nodes and from the 1st week in the spleens, respectively. The frequency of CD4+IL-17A+ T cells was significantly increased in the rejection group at the 3rd week (*P* ≤ 0.001) and then decreased at the 4th week, but there was no statistical difference in the lymph nodes between the survival group and the rejection group (*P* = 0.327) (Figures 3(g) and 4(g)).

### 3.3. The Imbalance of Th1/Th2 and Tregs/Th17 in Secondary Lymphoid Organs

There was no significant increase of the ratio of Th1/Th2 (CD4+IFN-*γ*+ T cells/CD4+IL-4+ T cells) in the lymph nodes and spleens in both the syngeneic and the allogeneic group. But the ratio in the allogeneic group was slightly higher than that in the syngeneic group in both the lymph nodes and spleens at the 2nd week. There was a significant increase of the Th1/Th2 ratio in the rejection group at the 3rd week in both the lymph nodes and spleens. The ratio of Th1/Th2 decreased both in the lymph nodes and spleens at the 4th week after corneal transplantation in the rejection group, but there was still a statistical difference in the spleens between these two groups (Figures 3(h) and 4(h)). In the first 2 weeks, the ratios of both Foxp3+Tregs/Th17 and LAP+Tregs/Th17 in the syngeneic group were higher than that in the allogeneic group in the lymph nodes and spleens. However, the ratios of Foxp3+Tregs/Th17 and LAP+Tregs/Th17 markedly deceased in the rejection group compared with survival group at the 3rd and 4th week (Figures 3(i), 3(j), 4(i), and 4).

### 3.4. CBA Analysis of Inflammation-Related Cytokines in the Aqueous Humor

**Figure 5:**
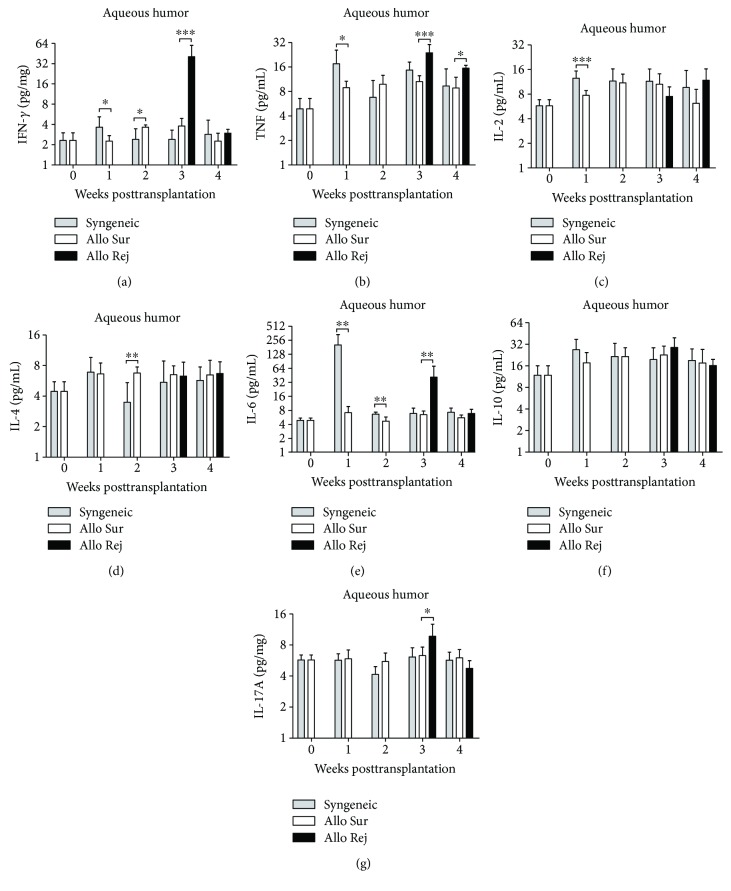
Comparison of the cytokines in the aqueous humor. (a–g) The concentration of IFN-*γ*, TNF, IL-2, IL-4, IL-6, IL-10, and IL-17A from the survival group (Allo Sur), rejection group (Allo Rej), and the syngeneic group (Syn) was analyzed by CBA. *n* = 5 in each group. The data are presented as the mean + SEM; ^∗^*P* < 0.05, ^∗∗^*P* < 0.01, and ^∗∗∗^*P* < 0.001.

The cytokine expression in different groups is shown in Figure 5. The level of IFN-*γ* increased from the 2nd week after allogeneic corneal transplantation and then returned to normal level gradually. The levels of IFN-*γ* and IL-17A in the rejection group were higher than those in the survival group at the 3rd week (*P* ≤ 0.001, *P* = 0.023), but there was no statistical difference between these two groups at the 4th week (*P* = 0.341, *P* = 0.091) (Figures 5(a) and 5(g)). There was a statistically significant difference of TNF level between the rejection group and the survival group at the 3rd and 4th week, but there was no difference of IL-2, IL-4, and IL-10 levels (Figures 5(b)–5(d) and 5(f)). The level of IL-6 in the syngeneic group was apparently higher than that in the allogeneic group in the first two weeks after corneal transplantation. Surprisingly, the level of the IL-6 in the rejection group was higher than that in the survival group at the 3rd week (*P* = 0.007) (Figure 5(e)).

## 4. Discussion

Although there were some previous publications about the corneal rejection process in allogeneic transplantation, the survival time of corneal graft was different in different reports and there was only few studies about immune imbalance in rejected and surviving mice till now. Chen et al. reported that 53% of allogeneic corneal allografts were rejected 7 days after transplantation [14], while Chen et al. showed rejection appeared in some grafts 2 weeks after corneal transplantation [15]. According to our result, the new blood vessels reduced or even subsided after suture removal 7 days after transplantation, and most allografts were rejected from 14 to 21 days after operation. If the opacity score of the allografts reached 2 or above and did not improve 7 days after transplantation, the corneal grafts were regarded as being rejected. Our data showed that 62.5% of the allografts survived in our observation period, which was consistent with most of other studies. A study showed that approximately 50% of allografts survived within 50 days when C57BL/6 corneal allografts were transplanted onto BALB/c [16]. In this study, firstly, we analyzed the role of LAP+Tregs in the survival and rejected mice after penetrating transplantation; secondly, we investigated the immune balance between Th1 and Th2 cells, LAP+Tregs, and Th17 cells as well, which played an important role in maintaining the immune-privileged status of corneal transplantation.

Corneal allograft rejection is a delayed-type hypersensitivity reaction (DTH), and CD4+ T cells are the predominant cell type in this process. In our study, we found that both Th1 and Th2 cells were involved in corneal allograft rejection. The frequency of CD4+IL-4+ T cells increased obviously in the lymph nodes of the rejection group 3 weeks after operation. IL-4 can induce eosinophilia gathering, inflammation, and exacerbate the rejection response [17]. Meanwhile, Beauregard et al. also proposed that there was synergistic Th1/Th2 collaboration in rejecting the allografts [18]. However, CD4+ Th lymphocyte differentiation produced a large number of CD4+IFN-*γ*+ T cells, which shifted the Th1/Th2 balance toward Th1 population, while inhibiting CD4+Foxp3+ T cell frequency. In our previous research, Xu et al. found that Foxp3, LAP, and GARP in CD4+ T cells could be detected in ipsilateral drainage lymph nodes and spleens, but no significant difference was observed in Foxp3, LAP, and GARP expression on CD4+ T cells among three groups [19]. Furthermore, in our research, the allotransplanted mice were divided into rejection group and the survival group and we found that the frequencies of Foxp3+Tregs, GARP+Tregs, and LAP+Tregs in the survival group were significantly higher than those in the rejection group in the ipsilateral drainage lymph nodes and spleens 21 days after transplantation. Although Chauhan et al. thought that the mean fluorescence intensity of Foxp3 in Tregs from the LN was significantly higher in the allograft survival mice than those in the allograft rejection mice, instead of their increased number of Tregs [20], most studies have suggested that the upregulation of the number of CD4+Foxp3+Tregs in LN could improve corneal allograft survival [21, 22]. In our study, although the changes of Tregs were subtle in the lymph nodes, we still believe that increasing the number of Tregs is of great significance in prolonging the survival time in mice after corneal transplantation, because the proportion of regulatory T cells in the peripheral lymph nodes is relatively small. The result from a similar study revealed that the percentage of CD4+Foxp3+Tregs increased only 2% in the lymph nodes; nevertheless, the survival of graft was also prolonged, which is consistent with our findings [23]. But more studies are required to confirm that such small changes can impact graft rejection or acceptance. Meanwhile, the expression trend of CD4+LAP+ T cells and CD4+GARP+ T cells was consistent between the rejection group and the survival group, which was consistent with Noyan's report showing GARP and LAP were also coexpressed on the same proportion of activated Tregs [24]. Besides that, we also found that the population of CD4+IL-17A+ T cells significantly increased, while Tregs decreased in the rejection group 21 days after operation. There was no difference in the level of Th17 cells between the rejection group and the survival group 28 days after operation. Chen et al. reported that Th17 cells played a disease-promoting role at the early stage of corneal allograft rejection [14]. Yin et al. reported that Th17 cells produced high level of IL-17A at late-term rejection period. From our observation, most allotransplanted mice began to develop corneal rejection from 14 to 21 days after operation. Therefore, our study confirmed that an increase of Th17 cell differentiation occurred mainly in the acute rejection stage of corneal transplantation. The increase of LAP+Tregs/Th17 ratio in the allograft mice also contributed to the inhibition of corneal allograft rejection.

Moreover, we analyzed the changes of cytokines in the aqueous humor from the survival group and the rejection group. IFN-*γ*, TNF, and IL-2 are proinflammatory cytokines which are secreted by Th1 cells. We found that the level of IFN-*γ* and TNF but not IL-2 significantly increased in the rejection group. Maier et al. also found IL-2 maintaining a lower level in the aqueous humor of patients following penetrating keratoplasty [25]. IL-4 and IL-6 are cytokines secreted by Th2 cells. Although there was no significant change in the level of IL-4, we found elevated IL-6 level in the aqueous humor in an endothelial immune reaction. This is in consistent with the findings by Flynn et al. [26] and Funding et al. [27] who demonstrated that increased IL-6 levels were the result of reaction to the rejection process. Meanwhile, IL-6 is capable of inducing the production of the proinflammatory cytokine IL-17A [13]. Thus, both IL-6 and IL-17 simultaneously increased in the rejected mice after operation. Yin et al. showed that IL-6 might be involved in the transition to an environment where IL-17A could affect the viability of corneal allograft directly [28]. In contrast, IL-10, one of the most important immunosuppressive cytokines secreted by Tregs, has been proven to favor allograft acceptance and has a close relationship with the allograft's function. Unexpectedly, we did not find significant increase in IL-10 level in mice from the survival group. A study from Wang et al. [23, 29] found that all-trans retinoid acid and rapamycin can promote allogeneic corneal graft survival in mice, but the statistical difference for the level of IL-10 was very small in the aqueous humor. However, the frequency of CD4+IL-10+ T cells in the lymph nodes and spleens increased at the stage of corneal allograft rejection. Therefore, the exact role of IL-10 in modulating immunological reaction in allogeneic corneal transplantation still needs to be confirmed by further studies.

In summary, our study showed a role of imbalance in Th1/Th2 as well as LAP+Tregs/Th17 in corneal allograft rejection. The next clear step is to modulate the balance between LAP+Tregs and Th17 cells and raise the frequency of circulating CD4+LAP+Tregs to prolong the survival of the allografts in corneal transplantation. More studies are required to comprehensively explore the therapeutic potential of this novel subset of regulatory T cells in immune tolerance.

## Figures and Tables

**Table 1:** Quantitative analysis of inflammatory cells in the survival and rejection groups.

Inflammatory cells (cells/*μ*m^2^)	Survival group (*n* = 5)	Rejection group (*n* = 5)	*P* value
3 weeks	0	12.0 ± 5.05	<0.001
